# Phylogenetic analysis and expression profiles of jasmonate ZIM-domain gene family provide insight into abiotic stress resistance in sunflower

**DOI:** 10.3389/fpls.2022.1010404

**Published:** 2022-10-04

**Authors:** Huifang Song, Xinxuan Fu, Juan Li, Tianzeng Niu, Jie Shen, Xi Wang, Yunling Li, Qinwen Hou, Ake Liu

**Affiliations:** ^1^Department of Life Sciences, Changzhi University, Changzhi, China; ^2^Key Laboratory of State Forestry and Grassland Administration on Tropical Forestry Research, Research Institute of Tropical Forestry, Chinese Academy of Forestry, Guangzhou, China

**Keywords:** sunflower, *JAZ*, phylogenetic analysis, hormone, abiotic stress, WGCNA

## Abstract

Jasmonate ZIM-domain (JAZ) proteins act as inhibitory factors of the jasmonic acid (JA) pathway, which is involved in regulating plant development and defense responses. However, there are no extensive studies available on *JAZ* genes in sunflower (*Helianthus annuus* L.). In this study, the phylogenetic analysis of 139 putative *JAZ* genes from eight plants demonstrated that these *JAZs* could be divided into five groups (Groups I–V), and the 27 sunflower *JAZs* (*HaJAZs*) were classified into these five groups. All groups contained genes from both monocotyledons and dicotyledons, indicating that the emergence of *JAZ* genes predates the differentiation of monocotyledons and dicotyledons. Both segmental and tandem duplications contributed greatly to this gene family’s expansion in sunflower, especially in Group II. Moreover, the expression profiles of *HaJAZ* genes under normal conditions, hormone treatments or abiotic stresses were analyzed based on RNA-seq data. *HaJAZ2* may be undergoing pseudogenization as a nonfunctional gene because it was not expressed in any tissue. Many *HaJAZ* genes in roots upregulated their expression when involved in responding to exogenous hormones, especially methyl-jasmonate. The abiotic stress treatments of sunflower showed that *HaJAZ5*, *HaJAZ15*, *HaJAZ17*, *HaJAZ20*, and *HaJAZ21* tend to be sensitive to certain abiotic stresses. *HaJAZs* from different groups may share similar functions but also exercise their unique functions when responding to abiotic stresses. We speculated that this gene family was conserved in sequence but varied in its expression among duplicated *HaJAZ* genes, which implies that they may confer neofunctionalization in the adaptation to abiotic stresses; this work provides insight into the resistance of sunflowers and their adaptation to diverse environmental conditions.

## Introduction

Phytohormones are the most important endogenous substances for modulating physiological and molecular responses and are thus essential for plant survival (Chini et al., 2007; Wolters and Jurgens, 2009). Among them, jasmonic acid (JA) is involved in plant responses to biotic or abiotic stresses, as well as growth and development (Garrido-Bigotes et al., 2019). The main components of the JA signaling pathway are the coronatine-insensitive 1 (COI1)-JAZ complex receptor and MYC transcription factors (TFs; Chini et al., 2007; Melotto et al., 2008; Chung et al., 2009). Among these components, the inhibitory factor JAZ (JA zinc-finger inflorescence meristem (ZIM)-domain), a key family protein of the JA pathway, comes from the TIFY family (Chung et al., 2009). The TIFY family is also known as ZIM (Vanholme et al., 2007) and can be further divided into four subfamilies (TIFY, ZML, JAZ, and PPD) based on the specific domain compositions (Bai et al., 2011; Zhang et al., 2012). Different subfamilies have varied domain distributions (Bai et al., 2011). For instance, the TIFY subfamily contains only the TIFY domain; the ZML subfamily contains the TIFY, CCT, and C2C2-GATA domains; the PPD subfamily contains TIFY domains but lacks CCT and GATA domains, while comprising N-terminal PPD domains; and the JAZ subfamily contains TIFY and Jas (also named CCT 2) domains.

JAZ proteins are repressor proteins and are the most extensively studied TIFY subfamily among plants. The JA signaling pathway can respond to JA stimulation, causing the binding MYC transcription factor to be released and initiating the transcription of downstream JA-response genes (Santner and Estelle, 2007). JAZ proteins can also bind to other proteins, thus linking the JA signaling pathway with other pathways and thereby affecting gene expression (Thines et al., 2007). Both the TIFY and Jas domains are required for JAZ proteins to function in the JA signaling pathway (Chini et al., 2007). When JA accumulates after exposure to environmental stimuli, this accumulation can accelerate the binding of the SCF^COI1^ complex to perceive JA-Ile and hence degrade JAZ proteins *via* 26S proteasomes. This process can induce the activity of TFs (such as MYC2) to regulate the expression of JA-responsive genes (Chini et al., 2009, Guo et al., 2018a). Therefore, the COI1–JAZ–MYC2 model has been regarded as the most pivotal signal module in the JA pathway. Additionally, JAZ proteins can also recruit TPL (TOPLESS) or TPR (TPL-related protein) repressors through the Novel INteractor of JAZ (NINJA) to repress the expression of TFs without stimulation or JA accumulation (Pauwels et al., 2010).

JAZ plays a key role in the response of plants to biotic and abiotic stresses. In Arabidopsis, *JAZ* can promote plant growth and development by preventing unrestricted metabolic processes (Guo et al., 2018b). In wheat, *JAZ* can regulate gene expression and inhibit seed germination (Ju et al., 2019). In rice, *OsJAZ1* can act as a transcriptional regulator to improve drought tolerance (Seo et al., 2011). *OsJAZ8* is involved in the induction of monoterpene linalool, which makes rice resistant to bacterial blight (Taniguchi et al., 2014). *OsJAZ9* is responsive to salt stress or potassium deficiency tolerance in rice (Wu et al., 2015; Singh et al., 2020). Overexpression of *OsJAZ8* can increase the salinity tolerance of transgenic seedlings (Peethambaran et al., 2018). *OsJAZ13* can suppress the expression of *MYC2* and *ERF1* to regulate cell death in a JA/ethylene-dependent manner and hence modulate the balance between defense and growth under environmental stresses (Feng et al., 2020). In addition, overexpression of *TaJAZ1* enhances the resistance of wheat to powdery mildew (Jing et al., 2019), and overexpression of *GhJAZ2* can also enhance the salinity tolerance of transgenic cotton (Sun et al., 2017). In *Triticum durum*, overexpression of *TdTIFY11a* can enhance salt tolerance (Ebel et al., 2018).

Sunflower (*Helianthus annuus* L.) is an annual plant from the family Asteraceae. It is one of the four major oil crops around the world and an important ornamental plant. Due to its strong resistance to abiotic stresses, sunflower can serve as an important genetic resource for determining plant resistance mechanisms and hence improving its resistance (Garrido-Bigotes et al., 2019). In this study, extensive analysis of the sunflower JAZ (HaJAZ) family was performed to understand their diversity, expansion, and evolutionary fate after duplications, as well as defense responses, which may lay a theoretical foundation for the further exploration of sunflower *JAZ* gene functions and provide a new perspective for sunflower resistance breeding.

## Results

### Twenty-seven *JAZ* genes identified in sunflower

In total, 27 *HaJAZ* genes were identified from the sunflower genome and named according to their physical positions on the chromosomes (Supplementary Figure S1). They were irregularly distributed on 17 sunflower chromosomes. For instance, chromosome 12 (Chr 12) had the highest density of *HaJAZ* genes (7 members), while only one *HaJAZ* gene was found on each of five chromosomes (Chr1, Chr2, Chr9, Chr16 and Chr17). Detailed gene information for each *HaJAZ* is listed in Supplementary Table S1, such as gene length, predicted molecular weight (MW) and isoelectric point (pI). Despite their close evolutionary relationship, HaJAZ proteins and their coding regions varied greatly in length. The protein lengths ranged from 124 to 345 amino acids, and the gene lengths ranged from 824 bp to 6,906 bp (Supplementary Table S1).

**Figure 1:**
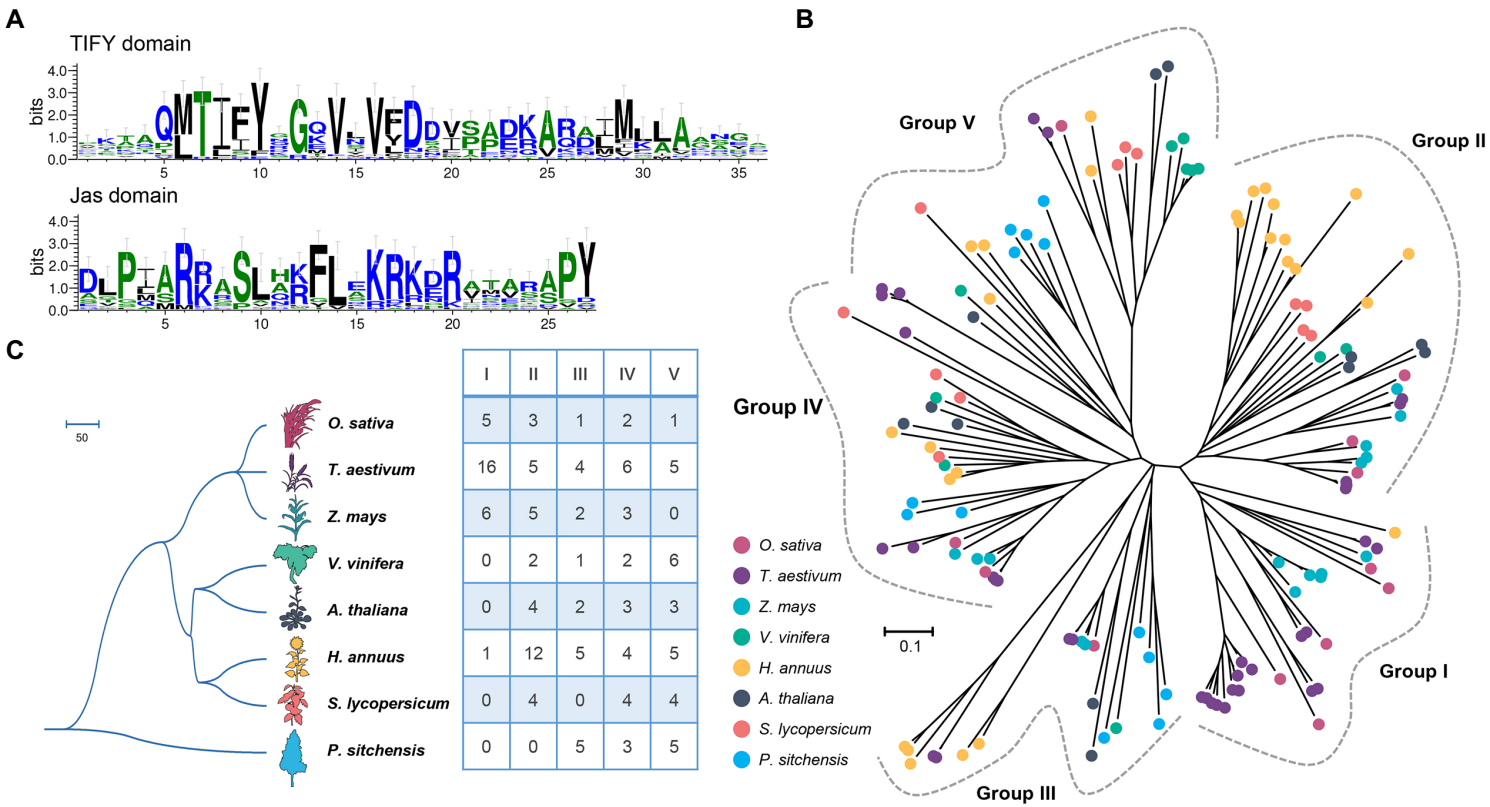
Conserved domains and phylogenetic analyses of the JAZ family in sunflower. **(A)** The consensus sequences for TIFY and Jas were generated with WebLogo. The numbers at the bottom indicate the positions of amino acid residues in each domain. The height of each letter is proportional to the frequency of the corresponding residue at that position, and the letters are ordered such that the most frequent residue is on top. **(B)** Phylogenetic tree of JAZ proteins derived from sunflower and seven other plants. Members belonging to the same species are presented with the same label. **(C)** Numeric comparisons of five group members. The species tree was obtained from TimeTree (http://www.timetree.org/).Theimages of *Arabidopsis thaliana*, *Solanum lycopersicum*, and *Helianthus annuus* were obtained from a previous study (Sucher et al., 2020).

For the peptides, two important functional domains were identified among the JAZ-family proteins, namely, TIFY and Jas domains. Figure 1A shows that the TIFY domain was less conserved but had a core TIFYXG motif with two adjacent sites, Val (V) 14 and V16. Moreover, the Gln (Q) 5, Asp (D) 18, Ala (A) 25, and Met (M) 29 sites in the TIFY domain of sunflower were also highly conserved, which was consistent with other species, such as *Arabidopsis*, rice, and maize (Kynast et al., 2001; Vanholme et al., 2007). In contrast, the Jas motif seems more conserved in sunflower, particularly at the Pro (P) 3, Arg (R) 6, Ser (S) 9, Phe (F) 13, Leu (L) 14, Lys (K) 16, and Arg (R) 20 sites (Figure 1A). Additionally, the sites Pro (P) and Tyr (Y) located at the C-terminus of the Jas motif were also conserved, which can serve as symbols to distinguish the Jas motif of the JAZ subfamily from the divergent motif of the PPD subfamily.

### Five groups defined among the *JAZ* genes of eight species

To reveal the phylogenetic relationships within the JAZ family among different plants, a phylogenetic tree containing JAZ proteins was reconstructed based on the alignment of conserved TIFY and Jas domain sequences derived from eight plants (Figure 1B), including sunflower (27), *Arabidopsis* (*Arabidopsis thaliana*, 12), rice (*Oryza sativa*, 12), maize (*Zea mays*, 16), wheat (*Tricicum aestivum*, 36), grape (*Vitis vinifera*, 11), tomato (*Solanum lycopersicum*, 12), and Sitka spruce (*Picea sitchensis*, 13) (Figure 1C). The evolutionary history of these proteins was inferred using the neighbor-joining (NJ) method implemented in Molecular Evolutionary Genetics Analysis (MEGA) 7.0. The tree with the optimal branch length sum (31.45) is shown in Figure 1B. The tree was drawn to scale, and the branch lengths are in the same units as the evolutionary distances used to infer the phylogenetic tree. All ambiguous positions were removed for each sequence pair, and 101 positions were considered in the final dataset.

According to the phylogenetic tree, all 139 JAZ members could be clustered into five groups (Figure 1B; Supplementary Figure S2, Groups I–V). Group II had the maximum member number (35) and the highest percentage of sunflower JAZ proteins (12/27). In this group, 10 *HaJAZs* clustered together to form one clade, possibly resulting from species-specific expansion. Moreover, the JAZs from dicotyledon plants formed a cluster sister to the genes derived from monocotyledonous plants in Group II. Group I had the lowest number of members and contained only one *HaJAZ*. Group I contained JAZ members from sunflower, maize, rice, and wheat; sunflower was the only dicotyledonous plant in this group. Among the five groups, only Group IV contained *JAZ* genes from all eight investigated species. The *JAZ* genes from Sitka spruce, the outgroup species in this study, were classified into Groups III–V; this may indicate that genes from these groups are more ancient than other genes. Overall, most groups contained both monocotyledons and dicotyledons, indicating that JAZ proteins are distributed among defined groups and that the emergence of *JAZ* genes predates the differentiation of monocotyledons and dicotyledons.

Moreover, we also examined the gene structures, motif and domain distributions of the *HaJAZ* family members (Supplementary Figure S3). It showed that the *HaJAZ* genes of the same group contained more similar exon-intron structures and motif compositions than those from different groups. Among the 10 identified conserved motifs, motifs 1 and 2 were contained in the Jas and TIFY domains, respectively, and were found in all *HaJAZ* proteins, which may regard as core motifs. All the HaJAZ members contained TIFY and Jas domains, and the *HaJAZ12*, *HaJAZ25* and *HaJAZ26* also contained a GATA-type zinc finger located at the C-terminal of the proteins. Totally, all these results can provide further evidence for our classification scheme based only on the results of the phylogenetic analysis.

### Tandem and segmental duplications contribute to the expansion of *HaJAZ* genes

**Figure 2:**
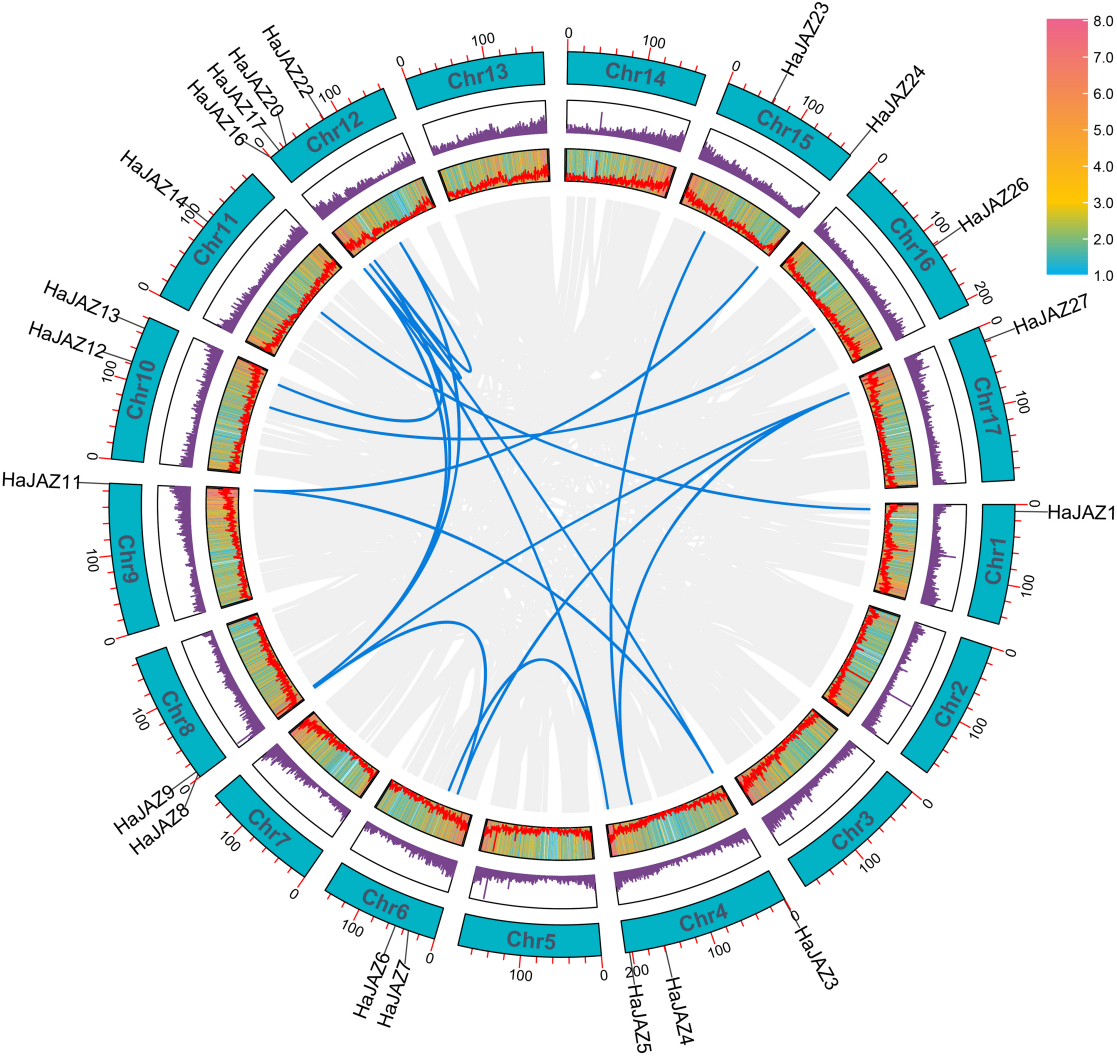
Schematic representation of the collinear relationships of *HaJAZ* genes. The blue and gray lines indicate the *HaJAZs* and all genes resulting from segmental duplications. The gene density on each chromosome is depicted by the heatmap next to each rectangle. Each chromosome is indicated with a turquoise rectangle, and the corresponding chromosome number is shown at the top of each chromosome.

To study the driving forces behind gene family expansion, two duplicated gene patterns were identified among JAZ family members in the sunflower genome, namely, tandem and segmental duplication. Apart from one tandem-duplicated pair (Supplementary Figure S1, *HaJAZ19* and *HaJAZ18*), the remaining 18 pairs were classified as segmental duplications (Figure 2). Among them, many duplicated genes may result from two rounds of duplication events occurring in a chromosome segment, such as *HaJAZ4*, *HaJAZ23*, and *HaJAZ27*. To further determine the extent of selection pressures among duplicated genes, we calculated the synonymous substitution rate (*d_S_*) and nonsynonymous substitution rate (*d_N_*) values for the 19 duplicated gene pairs. As shown in Supplementary Table S2, the *d_N_*/*d_S_* (ω) values ranged from 0.015 to 0.549, indicating that all the analyzed gene pairs experienced purifying selection. The tandem duplicated pair possessed the greatest ω ratio values, possibly indicating that these genes experienced more relaxed purifying selection. Additionally, three duplicated gene pairs (*HaJAZ9* vs. *HaJAZ16*, *HaJAZ20* vs. *HaJAZ16* and *HaJAZ4* vs. *HaJAZ27*) had much higher *d_S_* values than the other pairs (Supplementary Table S2). These findings may provide insight into the important contributions of segmental and tandem duplications to *JAZ* expansion in sunflower.

### Expression patterns of sunflower *JAZs* in various tissues

**Figure 3:**
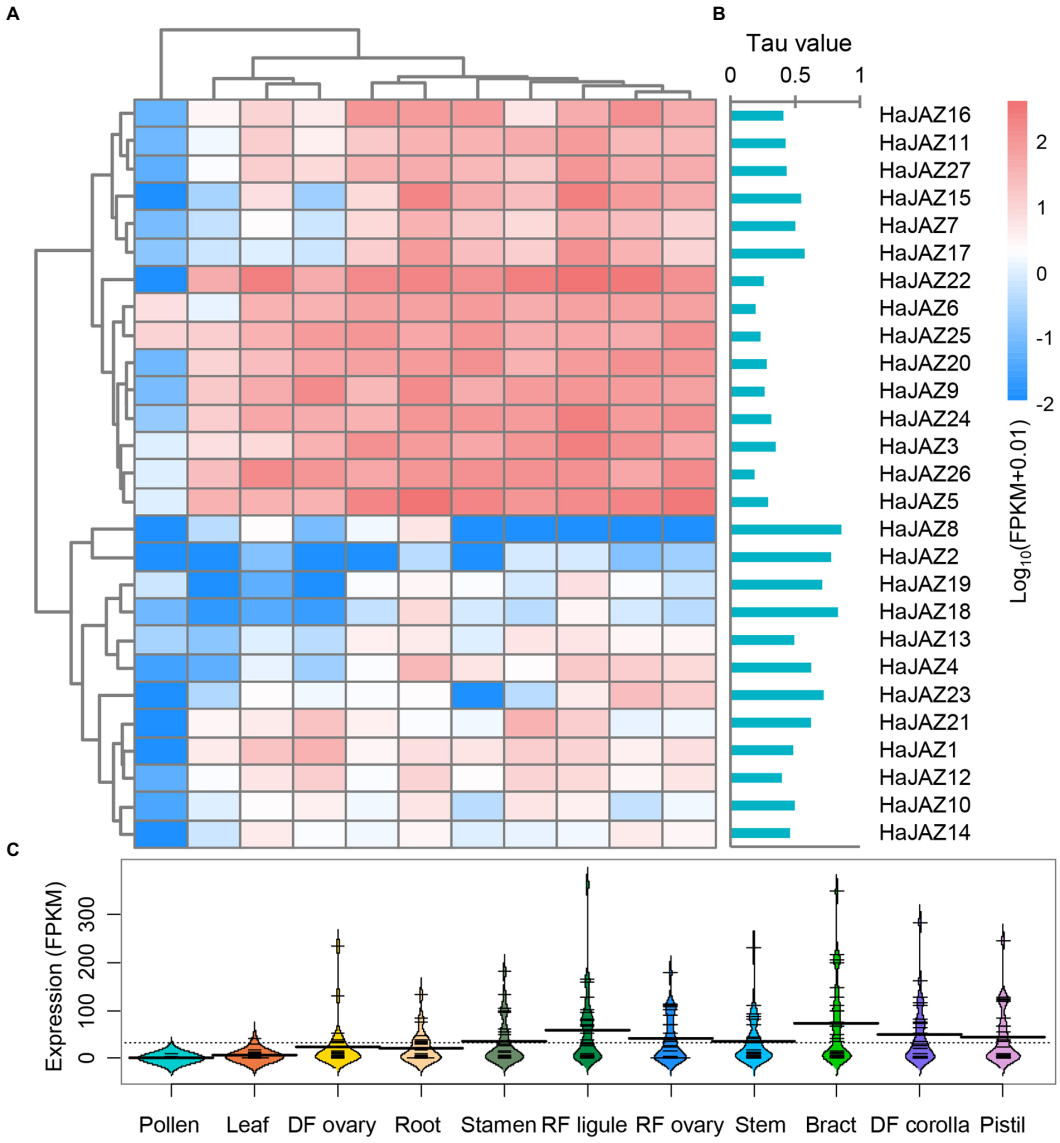
Expression profiles of *HaJAZ* genes in different tissues. **(A)** The normalized expression levels of the hierarchical clustering of 27 *HaJAZ* genes in 11 tissues. The relative expression levels corresponding to the log_2_-transformed FPKM values after adding a pseudocount of 0.01 are shown. The expression levels are indicated by a graded color scale from blue to red. **(B)** The *τ* values were calculated for *HaJAZs* to identify their tissue-specific expression. Low *τ* values correspond to widely expressed genes, while high values correspond to tissue-specific genes. **(C)** The expression values of all the *HaJAZ* genes in each tissue.

We analyzed the expression profiles of all *HaJAZs* in 11 different tissues, including leaf, root, bract, stem, stamen, pistil, pollen, RF (ray floret) ligule, RF ovary, DF (disc floret) corolla, and DF ovary tissues, using RNA-seq data obtained from a previous study (Badouin et al., 2017). As shown in Figure 3, both the hierarchical clustering analysis results and tau (*τ*) values showed that the expression patterns of *HaJAZ* genes could be divided into three clusters. First, more than half of the *HaJAZ* genes were highly expressed in the majority of the tested sunflower tissues. For example, *HaJAZ22* was highly expressed in sunflower stems, DF ovaries, stamens, bracts, and DF corollas. Second, the expression levels of some genes (such as *HaJAZ8*, *HaJAZ2*, *HaJAZ19*, and *HaJAZ18*) were low or not expressed. In particular, the expression of *HaJAZ8* and *HaJAZ2* was extremely low in almost all tissues. The expression levels of *HaJAZ19* and *HaJAZ18* in flower tissues, such as stamens, RF ligules, DF ovaries, bracts, and DF corollas, were higher than the corresponding expression levels in other tissues, indicating that these genes may play a role in flower development in sunflowers. Third, the transcript abundance of *JAZ* genes was low in the pollen [Figure 3C; only *HaJAZ6* and *HaJAZ25* had FPKM (fragments per kilobase of exon model per million mapped fragments) values >1].

### Expression patterns of *HaJAZ* genes in response to hormones

**Figure 4:**
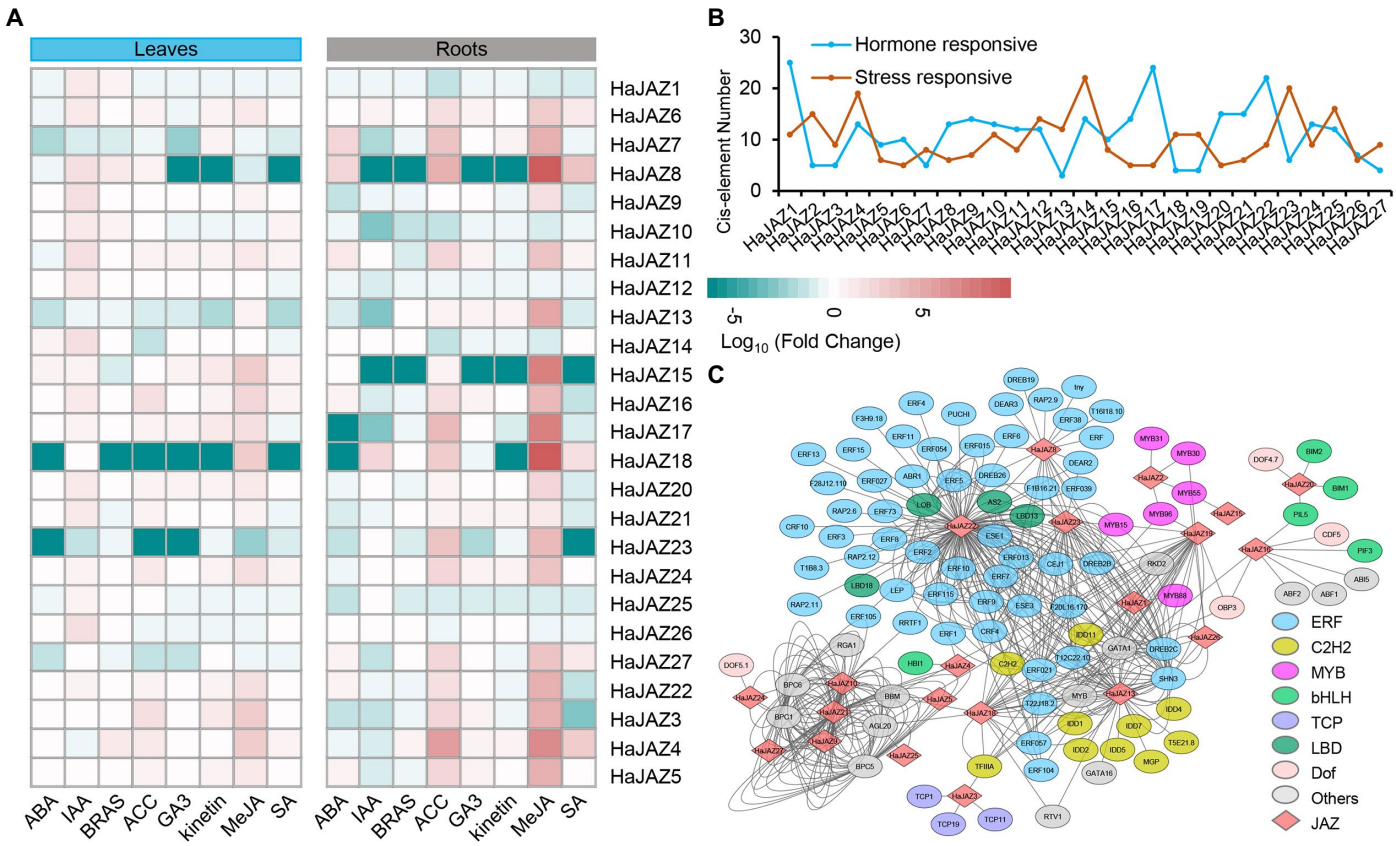
Expression changes in sunflower *JAZ* genes in response to different hormone treatments in the leaves and roots. **(A)** Log_10_-fold changes between treatments were used to present expression changes. The expression levels are illustrated by the graded color scale. **(B)** Two patterns of *cis*-elements, hormones, and salt-responsive elements, were predicted for the upstream 2 kb sequence of each *HaJAZ* gene. **(C)** The regulation network between *HaJAZs* and the potential TFs that bind to the HaJAZ promoters.

Accordingly, *JAZs* participate in the hormone signal transduction and crosstalk of plants, so it is necessary to study the expression patterns of *JAZs* under various exogenous hormonal treatments, including abscisic acid (ABA), β-indoleacetic acid (IAA), brassinosteroids (BRAS), 1-aminocyclopropane carboxylic acid (ACC), gibberellic acid 3 (GA3), kinetin, methyl-jasmonate (MeJA), and salicylic acid (SA). Figure 4A shows that *HaJAZ2* and *HaJAZ19* were not expressed in any of the treatment or control samples in either roots or leaves. The majority of *HaJAZs* expressed moderately upregulated expression under MeJA treatment in roots (such as *HaJAZ13*, *15*, *17*, and *4*), especially *HaJAZ8* and *HaJAZ18,* and several *HaJAZs* also had strong responses to ACC in roots. Furthermore, some genes in roots were involved in responding to other exogenous hormones, such as *HaJAZ8* and *HaJAZ7* to ABA, *HaJAZ4* to SA and *HaJAZ18* to IAA. These results indicated that the expression levels of *HaJAZs* were altered in response to various hormone inductions and were involved in the hormone signaling pathway in sunflower.

To explore the regulatory regions, we analyzed the *cis*-elements in upstream sequences of *HaJAZ* genes. Five hundred and seventy-six *cis*-elements of promoters related to environmental stress and hormone-responsive were detected (Figure 4B; Supplementary Table S3). It has been found that 21 *HaJAZs* (*HaJAZ1-3*, *HaJAZ5-6*, *HaJAZ8-12*, and *HaJAZ15-25*) had at least one JA-responsive element. Moreover, the promoter regions of 26 *HaJAZs* (except *HaJAZ3*) contain abscisic acid-responsive elements (ABREs). Sixteen *HaJAZs* contained auxin-responsive elements, 13 *HaJAZs* had gibberellin-responsive elements, and 10 *HaJAZs* contained salicylic acid-responsive elements (Supplementary Table S3). *HaJAZ7*, *HaJAZ13*, *HaJAZ23*, and *HaJAZ27* had JA- and abscisic acid-responsive elements, and their expression was downregulated greatly when treated with ABA, ACC, GA3, kinetin or SA in leaves. For *HaJAZ4*, *HaJAZ8*, *HaJAZ15*, *HaJAZ17*, and *HaJAZ23* in roots, their expression was downregulated greatly when treated with ABA, IAA, and BRAS, but the expression was upregulated greatly when treated with MeJA, especially *HaJAZ8* and *HaJAZ17* (Figure 4A). The hormone response elements of *HaJAZs* may play certain functions for their involvement in the hormone response.

We also predicted the potential TFs that bind to the *HaJAZ* promoters, and found that 102 TFs tend to bind to the regulatory regions of 22 *HaJAZ* genes (Figure 4C; Supplementary Table S4) and five *HaJAZs* have no predicted potential TFs (*HaJAZ6*, *HaJAZ7*, *HaJAZ11*, *HaJAZ14*, and *HaJAZ17*). The TFs involved in the regulation of *HaJAZ22* were the most abundant, and *HaJAZ5*, *HaJAZ12*, *HaJAZ15*, and *HaJAZ27* were the least abundant. Interestingly, different TF families have relatively strong binding tendencies to the different *HaJAZs*. For instance, 55 ERF TFs were mainly involved in the regulation of *HaJAZ22*, *HaJAZ13*, *HaJAZ8*, *HaJAZ18*, and *HaJAZ19* (Figure 4C). Among them, majority of the ERF TFs only bind to *HaJAZ22*. Moreover, C2H2 members centrally regulate *HaJAZ13* and coordinate the regulation of *HaJAZ13*, *HaJAZ18*, and *HaJAZ19* (Figure 4C). Additionally, the families such as TCP, bHLH, MYB, and LBD can also regulate some certain *HaJAZ* members.

### Expression patterns of *HaJAZ* genes in response to abiotic stresses

**Figure 5:**
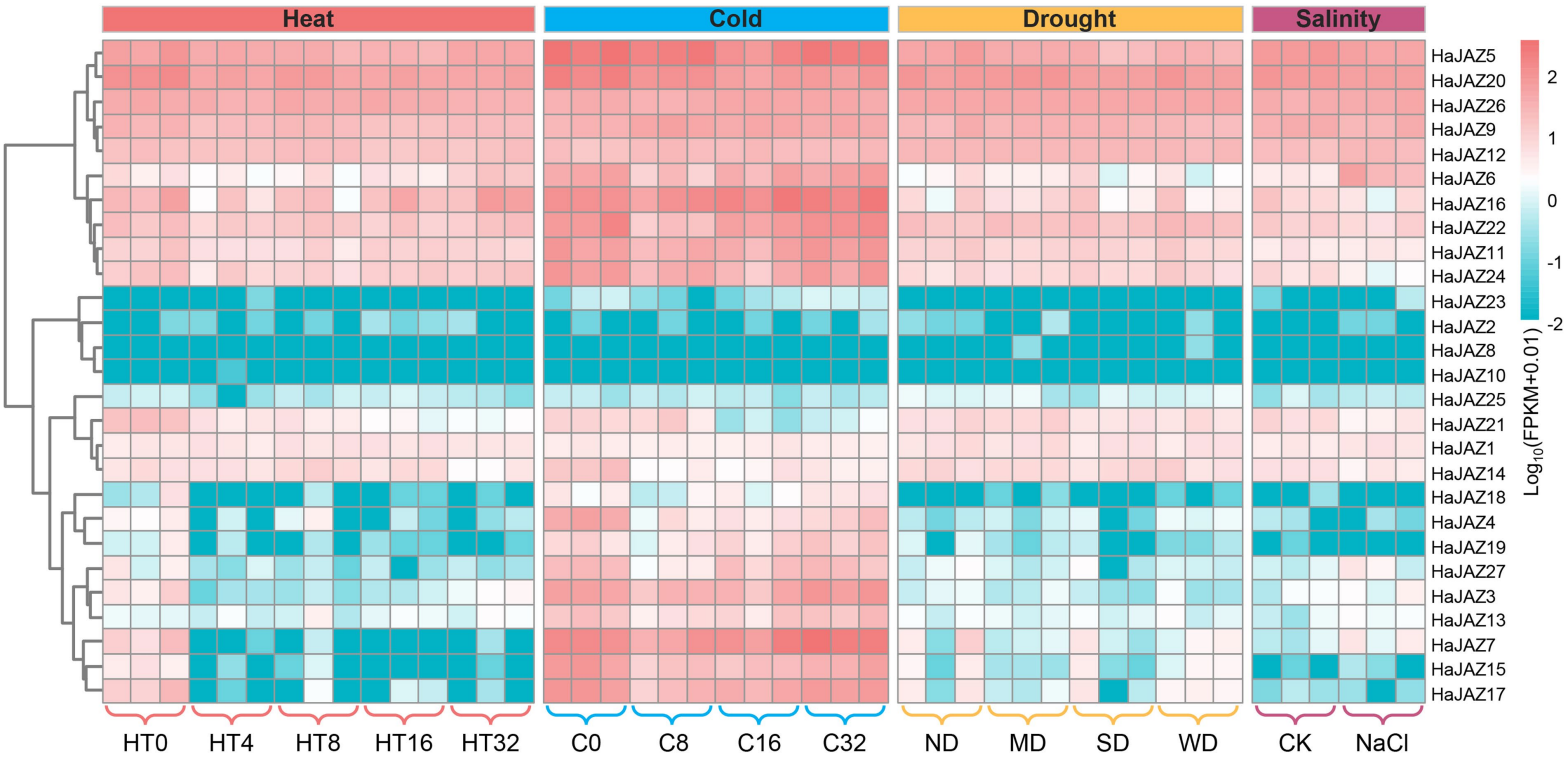
Expression profiles of *HaJAZ* genes in response to abiotic stress treatments. After sunflower seedlings grew to exhibit six true leaves, we selected uniformly growing seedlings and treated them at 39°C for 4, 8, 16, and 32 h. Seedlings not subjected to these temperature treatments were used as controls (HT0). For cold stress, seedlings with similar growth were selected and treated at 4°C for 8, 16, and 32 h (denoted C8, C16 and C32, respectively). Seedlings not subjected to this low-temperature treatment were selected as controls (C0). The different drought treatments included normal water supply (ND, no drought), mild drought stress (MD), severe drought stress (SD), and rehydration to normal water supply after 3 days of severe drought (WD). These water-control experiments commenced after the sunflower seedlings grew to exhibit new trifoliate leaves, and samples were taken after 5 days of drought stress. For the salinity stress experiments, 250 mmol L^−1^ NaCl was applied for 2 weeks. Seedlings not subjected to the NaCl treatment were selected as the controls (CK).

Plants are often subjected to multiple extreme environmental conditions during their growth periods, such as heat, drought, or other abiotic stresses. When under heat, cold, or drought stresses, the growth status of sunflower was not significantly affected. For example, the height of sunflower seedlings was not significantly different compared to those of the control group, and only the leaves showed mild wilting under severe drought, which also indicated that sunflower had strong abiotic stress resistance. Under salinity stress, the seedlings were shorter in height and the leaves wilted to some extent compared with those of the control group. To gain more insight into the role of *HaJAZ* genes in responding to abiotic stresses, we also analyzed the expression of *HaJAZ* genes under these four abiotic stress patterns. The results indicated that more than half of the genes exhibited significant expression level changes under these treatments. As shown in Figures 4B, 5, 11 *HaJAZ* genes were extremely temperature-sensitive (*HaJAZ3*, *HaJAZ4*, *HaJAZ7*, *HaJAZ14*, *HaJAZ15*, *HaJAZ17*, *HaJAZ19*, *HaJAZ20*, *HaJAZ21*, and *HaJAZ27*), and their expression levels were significantly downregulated following heat or cold treatment. The expression levels of three *HaJAZ* genes were consistently downregulated in the continuous high-temperature environment (*HaJAZ19*, *HaJAZ21* and *HaJAZ27*). When under cold treatment, the expression levels of *HaJAZ4*, *HaJAZ14*, *HaJAZ20*, and *HaJAZ21* were significantly reduced. *HaJAZ15* and *HaJAZ17* exhibited expression patterns that first decreased (at 8 h) and then rebounded (16 h − 32 h). In our prediction, eleven *HaJAZs* (*HaJAZ1-3*, *HaJAZ6*, *HaJAZ11*, *HaJAZ15*, *HaJAZ17*, and *HaJAZ22-25*) contained low-temperature responsive elements (Supplementary Table S3, LRT). Notably, only four genes were identified as differentially expressed genes (DEGs), and their expression levels were consistently downregulated following exposure to drought stress (*HaJAZ5*) or downregulated their expression in mild drought and then recovered somewhat in severe drought (*HaJAZ7*, *HaJAZ15*, and *HaJAZ17*), and all these genes were downregulated after drought stress and recovered following rehydration (Figure 5). However, twelve *HaJAZs* (*HaJAZ4*, *HaJAZ7-8*, *HaJAZ11-13*, *HaJAZ16*, *HaJAZ20*, *HaJAZ22-23* and *HaJAZ25-26*) contained drought response elements (Figure 4B, MBS). When exposed to salt stress, *HaJAZ16*, *HaJAZ21*, and *HaJAZ24* were significantly downregulated.

### Analysis of *HaJAZ* coexpressed genes in response to abiotic stresses based on WGCNA

**Figure 6:**
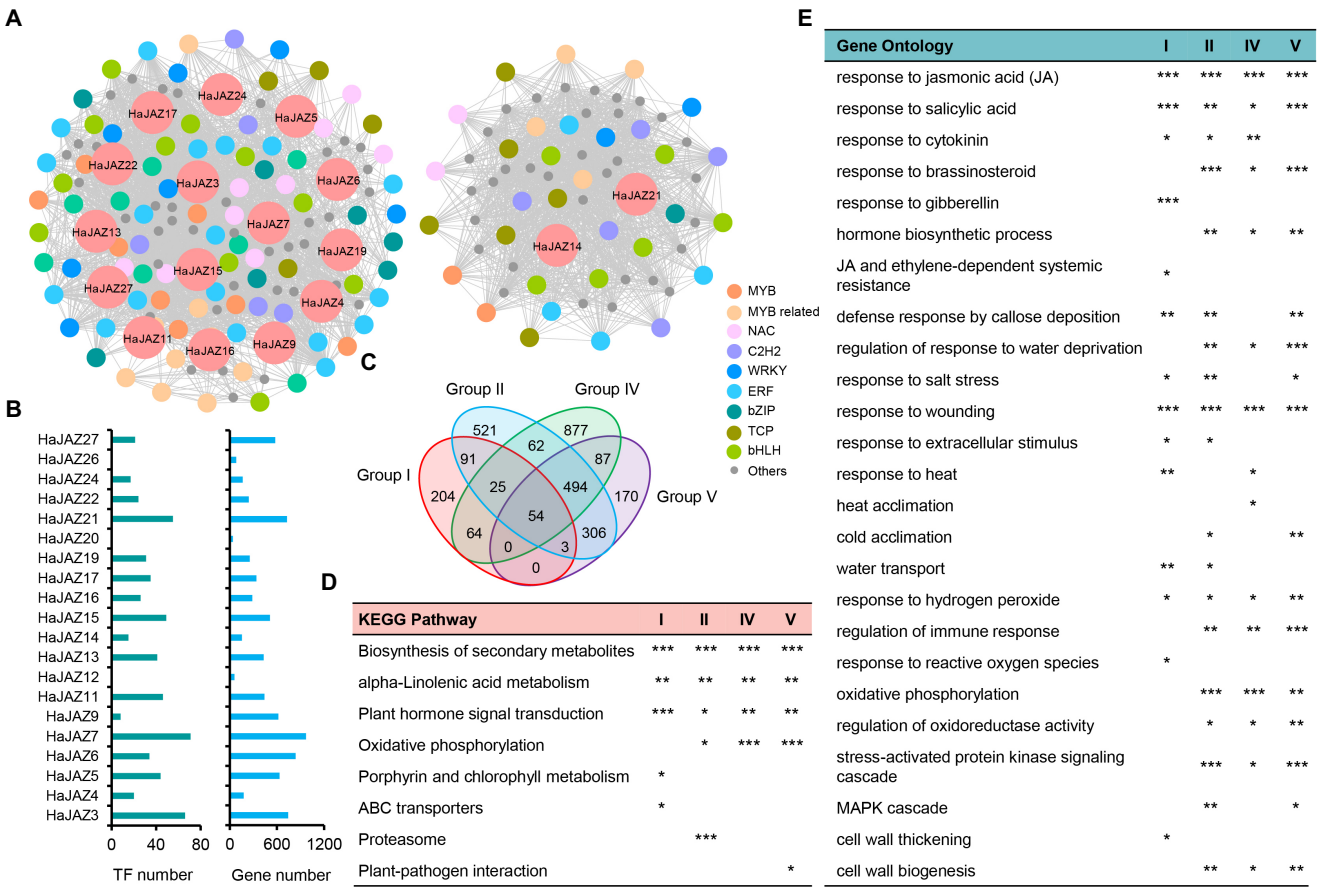
Functional enrichment of genes coexpressed with HaJAZ based on WGCNA. **(A)** The coexpression network for *HaJAZs* and their coexpressed genes. ERE: ethylene response elements; bHLH: basic/helix–loop–helix; MYB related; NAC: NAM, ATAF, and CUC; and bZIP: basic leucine zipper. **(B)** The numbers of those genes and TFs coexpressed with each *HaJAZ*. **(C)** Comparison of the coexpressed gene numbers of each JAZ group. **(D)** The *p*-values of significantly enriched KEGG pathways are shown. **(E)** The *p*-values of significantly enriched GO terms related to hormones, abiotic stress and the cell wall are presented here. ^*^p-value < 0.05; ^**^p-value < 0.01; ^***^p-value < 0.001.

To understand the roles of *HaJAZs* involved in abiotic stresses, we also conducted weighted correlation network analysis (WGCNA) based on these 45 samples under four abiotic stresses using our transcriptome data and found 34 modules. In total, 2,958 genes were coexpressed with 20 *HaJAZ* genes (Figures 6A–C). *HaJAZ3*, *HaJAZ6*, *HaJAZ7*, and *HaJAZ21* were associated with the greatest number of interacting genes. Among the coexpressed genes, 193 TFs were identified, and none of the TF members was found to be coexpressed with *HaJAZ12*, *HaJAZ20*, or *HaJAZ26* (Figure 6B). As shown in Figure 6A, two separate coexpression networks were constructed among these 17 *HaJAZ* genes and 193 TFs. Most TF families were ethylene-response elements (ERE), basic helix–loop–helix (bHLH) families (including 9 MYC2 members), MYB-related or NAM, ATAF, and CUC (NAC) families, with more than 10 members in each family. It is worth mentioning that five JA receptor *COI1* genes and nine *MYC2* genes were significantly correlated with 11 and 18 *HaJAZs* among these abiotic stresses (Supplementary Table S5, |*r*| > 0.7, *p*-value <0.01). The 11 *HaJAZs* were included in the 18 *HaJAZs*.

We further subdivided the coexpressed genes according to the classification of *HaJAZs* and found that there were 441, 1,556, 1,663, and 1,114 genes coexpressed with Groups I, II, IV, and V, respectively (Figure 6B) and only 54 genes were shared among them (Figure 6C). To study the potential functions of different JAZ groups, we conducted KEGG and GO functional enrichment analyses for these four gene lists. As shown in Figure 6D, all four lists were significantly enriched in biosynthesis of secondary metabolites, alpha-linolenic acid metabolism, and plant hormone signal transduction. These genes coexpressed with II, IV and V were also enriched in oxidative phosphorylation. The genes coexpressed with Group I were also enriched in porphyrin and chlorophyll metabolism and ABC transporters; those with Group II were enriched in proteasome, and those with Group V were enriched in plant–pathogen interaction. From the GO annotation (Figure 6E), all four lists were significantly enriched in response to JA and SA, wounding, and hydrogen peroxide. Groups I, II, and V were also enriched in response to salt stress and defense response by callose deposition. Groups II, IV, and V may be involved in the response to brassinosteroids, water deprivation, hormone biosynthetic processes, regulation of the immune response, oxidative phosphorylation, stress-activated protein kinase signaling cascades, and cell wall biogenesis. However, these genes coexpressed with different groups can be involved in different biological processes. For instance, Group I was enriched in response to gibberellin, reactive oxygen species, and cell wall thickening, and Group IV was enriched in heat acclimation. These results demonstrated that *HaJAZs* from different groups may share similar functions but also exercise their unique functions when responding to abiotic stresses.

## Discussion

Plant adaptations to environmental alterations generally rely on signaling networks. As important negative regulators impeding the JA response, JAZs have been demonstrated to play multiple roles in the growth, defense, and response to abiotic stress of plants (Garrido-Bigotes et al., 2019; Gupta et al., 2021). It has been reported that sunflower exhibits outstanding resistance to abiotic stresses (Škorić, 2016). However, limited information about the JAZ gene family in sunflower is currently known, thus hampering studies focused on resistance mechanisms. The well-annotated sunflower genome information (Badouin et al., 2017) and transcriptome technology provide fundamental materials for exploring the evolutionary relationships and expression diversity associated with the JAZ family in sunflower.

Previous studies have shown that *JAZ* genes can be divided into three to five groups (Xie et al., 2019; Shen et al., 2020). Combined with the JAZ proteins from seven other plants (Zhang et al., 2012; Chini et al., 2017; Xie et al., 2019; Han and Luthe, 2021), we classified these genes into five groups (Figure 1). The 27 *HaJAZ* genes were classified into five groups (Supplementary Table S1). Accordingly, plant genomes are less restricted in terms of genome size and chromosome number evolution, which leads to plants generally evolving more rapidly than other eukaryotes (Kejnovsky et al., 2009). Hence, the JAZ numbers varied among the tested species (Figure 1C). For instance, sunflower has twice as many *JAZs* as *A. thaliana*, which may result from the two additional genome-wide duplication event rounds that occurred during the sunflower evolution process (Badouin et al., 2017). Moreover, many *JAZ* genes undergo species-specific expansion, such as wheat, grape, and sunflower expansion (Figure 1). The phylogenetic and gene duplication analyses further indicated that genetic diversity and redundancy were present in this rapidly evolving gene family.

The sequestration of plants means that they are subject to greater survival risks than other eukaryotes and that they must rely on their genomic plasticity and biochemical changes to cope with different risks (Kejnovsky et al., 2009). The duplicated genes allow plants to enhance their adaptation to environmental stresses and promote plant differentiation (Leitch and Leitch, 2008). Tandem and segmental duplications are the main driving forces behind gene family expansion and gene functional differentiation in plants (Cannon et al., 2004). Among *HaJAZ* genes, segmental duplicates accounted for the main duplication pattern associated with *HaJAZ* gene amplification (Figure 2, 20/27), while the tandem duplicated gene pairs tended to experience relaxed purifying selection (Supplementary Table S2). After duplications, genes generally tend to pseudogenize or undergo neofunctionalization to breakdown functional redundancy to escape obsolescence and become fixed in the genome (Birchler and Yang, 2022). In this study, *HaJAZ2* tended to be not expressed under any condition, which may imply that it is undergoing pseudogenization, while other genes (*HaJAZ1* and *HAJAZ14*) tended to be divergently expressed among tissues or stress treatments (Figures 3–5).

Accordingly, *JAZ* genes play important roles in plant growth and stress responses (Li et al., 2014). We found that more than half of *HaJAZs* were highly expressed in most tissues, especially in the floral organs of sunflower plants (Figure 3), suggesting that JAZ family genes are involved in flower induction and stamen development (Kim et al., 2011). However, many *HaJAZ* genes were expressed at extremely low levels in the pollen (Figure 3C). In fact, many gene families, such as the WRKY and bHLH families, have been shown to be expressed at lower levels in sunflower pollen than in other tissues (Liu et al., 2020; Li et al., 2021). This information suggests that pollen may play a less important role than other tissues in responding to environmental stresses. Under MeJA treatment, we found that most *HaJAZ* genes showed significantly upregulated expression, especially *HaJAZ8* and *HaJAZ19* (Figure 4). Moreover, most *JAZ* genes in sunflower leaves were sensitive to IAA in the hormone treatment, and the *HaJAZ* genes in sunflower roots were highly regulated by MeJA and ACC (Figure 4). These results indicate that JAZ genes may play important roles not only in the JA signaling pathway but also in other hormone activities, such as the activities of IAA and ACC, which was shown in a previous study (Ullah et al., 2018).Previously, salinity stress can trigger the activation of the JA signaling pathway followed by the inhibition of cell elongation in the elongation zone, and salt-inhibited root growth partially involves the JA signaling pathway in *Arabidopsis* (Valenzuela et al., 2016). In our study, cold and salinity stress also downregulated the expression of *HaJAZs* (Figure 6). Moreover, many *HaJAZ* genes tended to downregulate their expression under the abiotic stresses. From the above results, we speculate that the sunflower *JAZ* gene plays a “repressor” role in response to different abiotic stresses.

COI1 can recruit JAZ family repressors to destroy the downstream MYC2 to regulate plant growth, development, and defense (Pieterse et al., 2014). In our study, the expression of JA receptor *COI1* genes was significantly negatively correlated with 11 *HaJAZs*, and nine *MYC2* genes were significantly positively correlated with 18 *HaJAZs* among these abiotic stresses (Supplementary Table S5). This result may indicate that these genes were involved in the response to abiotic stress through the JA pathway. Moreover, the functional analysis showed that all the coexpressed genes were enriched in response to SA and JA (Figure 6E). Accordingly, the SA and JA pathways act antagonistically on each other and provide the plant with a mechanism to fine-tune its defense response depending on the lifestyle of the enemy (Pieterse et al., 2014). Our data showed that the coexpressed genes of *HaJAZs* are involved in the biosynthesis of secondary metabolites, alpha-linolenic acid metabolism, and plant hormone signal transduction (Figure 6D). This can be linked with the fact that JA is biosynthesized from linolenic acid in chloroplast membranes (Sharma and Laxmi, 2015), and many secondary metabolites contribute greatly to plant environmental adaptation (Xu et al., 2022). Additionally, the enrichment analysis for each group demonstrated that different groups of *HaJAZs* may share similar functions but also exercise their unique functions when responding to abiotic stresses (Figures 6D,E).

**Figure 7:**
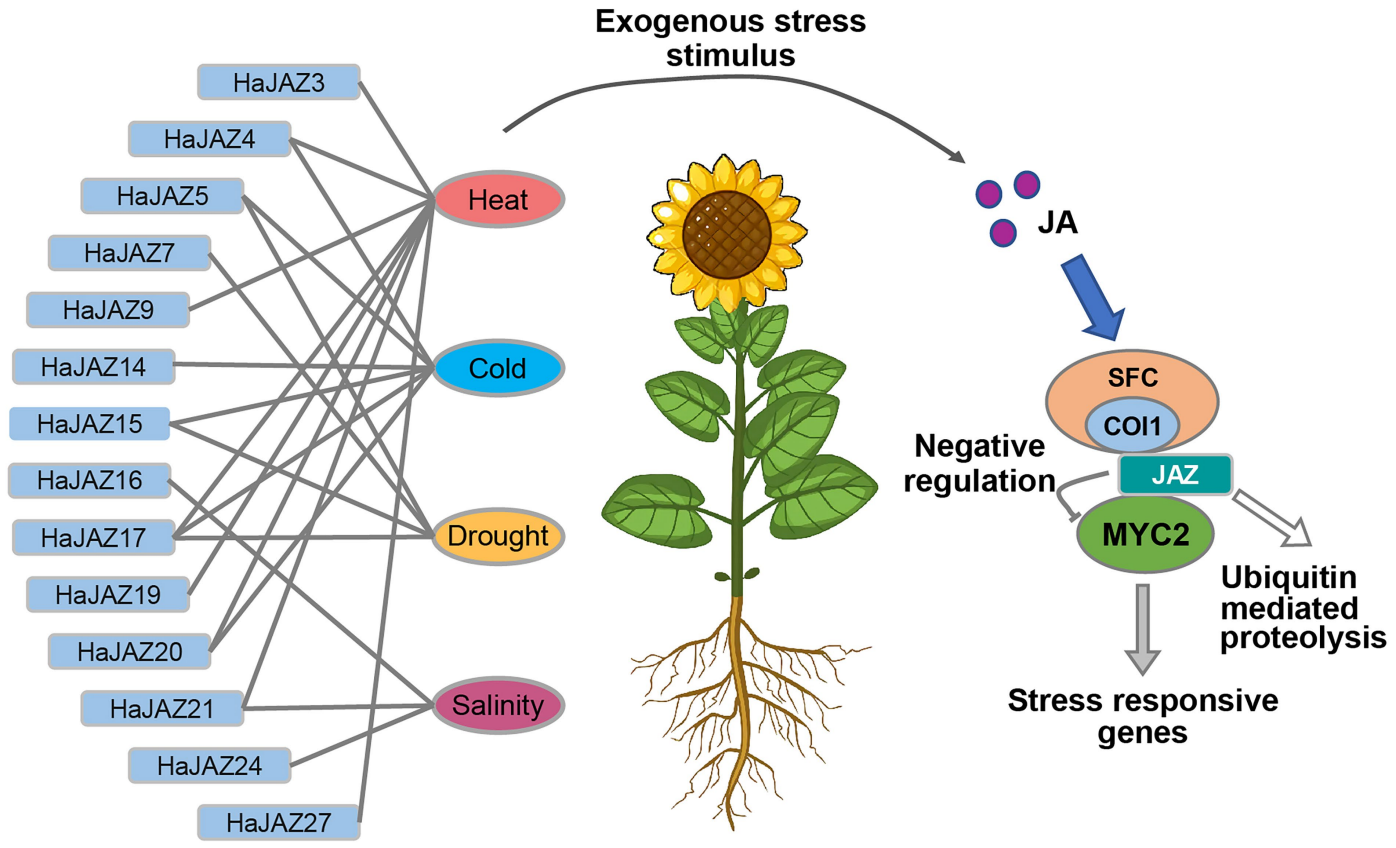
Model of *HaJAZ* genes regulating abiotic stresses. JA, jasmonic acid; COI1, coronatine-insensitive protein 1; SFC, SKP1 + Cdc53/cullin+Rbx1 + F-box protein complex; MYC2, Myeloid tissue proliferative protein in plants. The abbreviation name for each sample is shown in Figure 6.

In summary, 27 *HaJAZ* genes were identified, and some potential functions of the *HaJAZ* gene family and their phylogenetic information were revealed through bioinformatics analyses. This study found that *HaJAZ5*, *HaJAZ15*, *HaJAZ17*, *HaJAZ20*, and *HaJAZ21* tend to be sensitive to certain abiotic stresses, and the *JAZ* gene plays an irreplaceable role in plant abiotic stress responses (Figure 7). When sunflower is exposed to an exogenous stress stimulus, HaJAZ downregulates their expression or degrades their abundance by ubiquitin-mediated proteolysis. Then, the MYC2 TFs were released to activate the expression of downstream stress-responsive genes. We speculated that this gene family was conserved in sequence but varied in their expression among duplicated *HaJAZ* genes, indicating that they may gain environmental resistance through neofunctionalization. Our results may provide insight into the resistance of sunflower plants to diverse environmental conditions.

## Materials and methods

### Retrieval of *JAZ* genes

All sunflower protein sequences were obtained from the National Center for Biotechnology Information (NCBI) database. Hidden Markov model (HMM) profiles (El-Gebali et al., 2019) containing the TIFY (PF06200) and Jas (PF09425) domains were obtained from the Pfam database^1^ and used to identify potential *JAZ* genes in the sunflower genome with HMMER 3.2.1 software,^2^ with an *E*-value threshold of 10^−2^ (Prakash et al., 2017). Only the nonredundant proteins predicted by the online Simple Modular Architecture Research Tool (SMART)^3^ (Letunic and Bork, 2018) were identified as *HaJAZ* protein members. The JAZ protein sequences of *Arabidopsis*, rice, maize, wheat, grape, tomato, and Sitka spruce were derived from previous studies (Zhang et al., 2012; Chini et al., 2017; Xie et al., 2019; Zheng et al., 2020; Han and Luthe, 2021). The Expert Protein Analysis System (ExPASy) proteomics server^4^ was used to predict the different physicochemical parameters of the proteins (e.g., their molecular weight, theoretical isoelectric point, and number of amino acid residues).

### Sequence alignments and phylogenetic analyses

The JAZ protein sequences of sunflower, Arabidopsis, rice, maize, wheat, grape, and tomato were aligned using T-Coffee^5^ (Di Tommaso et al., 2011). An NJ phylogenetic tree was reconstructed using MEGA 7.0 (Kumar et al., 2016). The evolutionary distances were computed using the Poisson correction method in units of the number of amino acid substitutions per site. The other parameters were set as follows: 1,000 bootstrap replicates, pairwise deletions, and other default parameters (Kumar et al., 2016). The Weblogo3 application^6^ was used to visualize and analyze the conserved domains.

### *Cis*-element prediction of *HaJAZ* genes

The intron/exon structure of the sunflower *JAZ* genes was described using TBtools (v1.0692; Chen et al., 2020) based on comparisons of the predicted coding sequences (CDSs) derived based on genome annotation (Badouin et al., 2017). The online multiple Em for Motif Elicitation (MEME, http://meme-suite.org/) program was used to analyze the conserved motif structures of the proteins encoded by the sunflower JAZ genes. The two kbp-upstream sequences of the 27 *HaJAZ* genes beginning from the start codon ATG were extracted from the sunflower genome and regarded as the regulatory promoter regions. The *cis*-elements were analyzed using the online software PlantCARE database^7^ (Lescot et al., 2002). The potential TFs binding to the *HaJAZ* promoters were predicted through Plant Transcriptional Regulatory Map (PlantRegMap; Tian et al., 2020) and visualized with Cytoscape v.3.8.2 (Otasek et al., 2019).

### Chromosomal location and duplication of *HaJAZ* genes

The physical positions of the *HaJAZ* genes were obtained from the sunflower annotation file deposited in the NCBI database. We used TBtools (v1.0692) software (Chen et al., 2020) to derive the chromosomal locations of the *HaJAZ* genes. These genes were renamed according to their physical locations on individual chromosomes. To identify the duplication patterns of *HaJAZ* genes, MCScanX (Wang et al., 2012) was used under default settings to identify both segmental and tandem duplicated gene pairs encoded in the sunflower genome. The synonymous (*d_S_*) and nonsynonymous (*d_N_*) mutation rates were estimated using the online tool PAL2NAL (v14)^8^ (Suyama et al., 2006). The selection pressure of each duplicated gene pair was determined using the ω ratio.

### Expression patterns of *HaJAZ* genes in various tissues and under hormone treatment

To investigate the potential functions of *HaJAZ* genes in different tissues of sunflower under normal conditions, we acquired transcriptomic raw data of 11 tissues from a previous study (Badouin et al., 2017; Sequence Read Archive (SRA): SRP092742). These data represented pollen, stamen, pistil, DF ovary, DF corolla, bract, RF ovary, RF ligule, leaf, stem, and root tissues. We conducted reanalyses of the collected data following Liu et al. (2020). These data were log_2_-transformed and used to represent the expression profiles of *JAZ* genes through the pheatmap package implemented in R 3.6.3. To determine the tissue expression specificity, we introduced the parameter *τ* (Yanai et al., 2005) to assess whether a given gene was expressed in a tissue-specific manner. To avoid computing tissue specificity too sensitively, we added a penalty constant in the formula according to a previous study. The *τ* values were calculated as follows for each gene among different tissues: (Liu et al., 2020).


$$\tau =\frac{\sum_{i=1}^{n}\left(1-\hat{x_{i}}\right)}{n-1};\;\hat{x_{i}}=\frac{x_{i}}{\underset{1\leq i\leq n}{\max}\left(x_{i}\right)}$$

where *x_i_* is the expression of the gene in tissue *i* and *n* is the number of tissues. The FPKM values were normalized by performing log_2_ transformations after adding 1 to prevent the occurrence of negative values. The *τ* values ranged from 0 to 1, indicating that the gene expression specificity types ranged from widely expressed to specifically expressed.

To investigate whether *HaJAZ* genes play a role in hormonal responses in sunflower, data representing two tissues (roots and leaves) treated with 8 different hormones, ABA, IAA, BRAS, ACC, GA3, kinetin, MeJA, and SA, were obtained from a previous study (Badouin et al., 2017). All the transcriptome data obtained herein were analyzed following our previous study (Liu et al., 2020), and the expression fold change (FC) was derived for each of the treatments compared to the control.

### Plant materials and abiotic stress treatments

Heat, drought, cold and salinity stress experiments were carried out with the sunflower cultivar MH8361. The plants were grown under normal culture conditions following the above method until the sunflower seedlings grew to express six true leaves. For the heat and drought stress experiments, the treatments were conducted following the methods outlined in our previous study (Niu et al., 2022). For the cold stress experiment, seedlings with similar growth characteristics were selected and treated at 4°C for 8, 16, and 32 h (denoted C8, C16, and C32, respectively). Seedlings that were not subjected to the low-temperature treatment were selected as the control (C0). For the salinity stress experiment, 250 mmol L^−1^ sodium chloride (NaCl) was applied for 2 weeks. Seedlings not exposed to the NaCl treatment were selected as the control (CK). Each treatment was performed in triplicate. After the treatments were complete, the sunflower leaves were immediately collected and frozen in liquid nitrogen and then stored at −80°C until further use.

### RNA-seq and coexpression analyses of sunflower under various abiotic stresses

Total RNA was obtained from the above-mentioned samples using a Total Miniprep Kit following the manufacturer’s instructions (Axygen Bioscience, US). Then, 1 μg of RNA was reverse transcribed in total using PrimeScript RT Master Mix (TaKaRa) to synthesize cDNA. After constructing the cDNA library, polymerase chain reaction (PCR) amplification was conducted to enlarge the library fragments. Library selection was performed according to the fragment size (~450 bp). We then detected the total and effective concentrations using an Agilent 2,100 BioAnalyzer. For the qualified samples, high-throughput sequencing technology was adopted using the Illumina HiSeq™ 2000 sequencing platform, and paired-end (PE) sequencing was performed on these libraries.

After processing the RNA-Seq raw data using the FastQC and Cutadapt programs,^9^ the resulting high-quality reads were further mapped to the sunflower reference genome using HISAT2^10^ (Kim et al., 2019). The expression levels were normalized to fragments per kilobase of exon model per million mapped fragments (FPKM) to quantify the expression of each gene. Genes with |log_2_ FC| criteria >1.0 and *p*-values < 0.05 were regarded as differentially expressed genes (DEGs) between any two groups derived using DESeq^11^ (Wang et al., 2010).

Weighted gene coexpression networks were constructed using the WGCNA (v1.69) package in R (Langfelder and Horvath, 2008). All 14,610 genes derived from 45 samples were used to construct a signed coexpression network. The soft thresholding power (β) of 26 was selected to cause the networks to exhibit approximately scale-free topological conditions. Afterward, the adjacency matrix was transformed into a topological overlap matrix (TOM) to calculate the corresponding dissimilarity. All genes were hierarchically clustered using the topological overlap-based dissimilarity measure, and a gene dendrogram was generated based on TOM. The gene expression profile of each module identified from the gene dendrogram was calculated to test its association with each plant effective component. In the coexpression network, the edge weight (ranging from 0 to 1, corresponding to the interaction strength) of any two connected genes was determined based on their topology overlap measure. The networks were visualized using Cytoscape v.3.8.2 (Otasek et al., 2019). All statistical analyses and significance tests were performed using R project version 3.6.3.

## Data availability statement

The original contributions presented in the study are publicly available. This data can be found here: NCBI, PRJNA869183.

## Author contributions

AL and HS conceived the study. HS, XW, and JS performed the experiments. HS, XF, and AL contributed to the data analysis, bioinformatics analysis, and manuscript preparation. XF, JL, YL, and QH collected the public dataset of the studied species. All authors have read and approved the final version of the manuscript.

## Funding

This study was supported by the Scientific and Technological Innovation Programs of Higher Education Institutions in Shanxi (2021L514 and 2020L0620), and the National Natural Science Foundation of China (31901953). The funders had no role in the study design, data collection and analysis, decision to publish, or preparation of the manuscript.

## Conflict of interest

The authors declare that the research was conducted in the absence of any commercial or financial relationships that could be construed as a potential conflict of interest.

## Publisher’s note

All claims expressed in this article are solely those of the authors and do not necessarily represent those of their affiliated organizations, or those of the publisher, the editors and the reviewers. Any product that may be evaluated in this article, or claim that may be made by its manufacturer, is not guaranteed or endorsed by the publisher.

## Footnotes

^1^ http://pfam.xfam.org/

^2^ http://hmmer.janelia.org/

^3^ http://smart.embl-heidelberg.de/

^4^ https://web.expasy.org/compute_pi

^5^ https://tcoffee.crg.eu/apps/tcoffee/index.html

^6^ http://weblogo.berkeley.edu/logo.cgi

^7^ http://bioinformatics.psb.ugent.be/webtools/plantcare/html/

^8^ http://www.bork.embl.de/pal2nal/

^9^ http://cutadapt.readthedocs.io/en/stable/

^10^ http://ccb.jhu.edu/software/hisat2/index.shtml

^11^ https://www.bioconductor.org/packages//2.10/bioc/html/DESeq.html

## Supplementary material

SUPPLEMENTARY FIGURE S1 Chromosomal locations of sunflower JAZ genes. The HaJAZ members are named according to their physical position on the chromosomes.

SUPPLEMENTARY FIGURE S2 The tree was constructed using amino acid sequences under neighbor-joining (NJ) methods in MEGA 7.0. The numbers on the nodes indicate the bootstrap values derived from 1,000 replicates performed to test the reliability; values <70% are not shown.

SUPPLEMENTARY FIGURE S3 Conserved gene structures, motif and domain distribution of the sunflower JAZ gene family. The gene structure graph was drawn based on the genome annotation results. The MEME online tool was used to predict the motifs and identify the ten motifs present in the JAZ protein sequence. The domain distributions were predicted with the SMART online tool.

SUPPLEMENTARY FIGURE S4 The expression profiles of COI1 and MYC2 genes in response to abiotic stress treatments. The abbreviation name for each sample is shown in Figure 6E.

SUPPLEMENTARY TABLE S1 Detailed information on the sunflower JAZ gene family.

SUPPLEMENTARY TABLE S2 Synonymous and nonsynonymous substitution rates and Pearson correlation coefficient of expression for the duplicated gene pairs among sunflower JAZ genes. N, number of nonsynonymous sites; S, number of synonymous sites; dN, nonsynonymous substitution rate; and dS, synonymous substitution rate. The dS and dN values were calculated using codeml in the PAML package.

SUPPLEMENTARY TABLE S3 The cis-element patterns of each HaJAZ gene predicted for two kbp-upstream sequences from the start codon.

SUPPLEMENTARY TABLE S4 The potential TFs binding to the promoters of HaJAZs predicted through PlantRegMap.

SUPPLEMENTARY TABLE S5 Correlation test between the expression levels of COI1 genes, MYC2 genes and HaJAZ genes.
