# Supplementary figures and images for: Phylogenetic analysis and expression profiles of jasmonate ZIM-domain gene family provide insight into abiotic stress resistance in sunflower
Source: Front Plant Sci. 2022 Oct 4;13:1010404. doi: 10.3389/fpls.2022.1010404 (PMC9580003; doi:10.3389/fpls.2022.1010404)

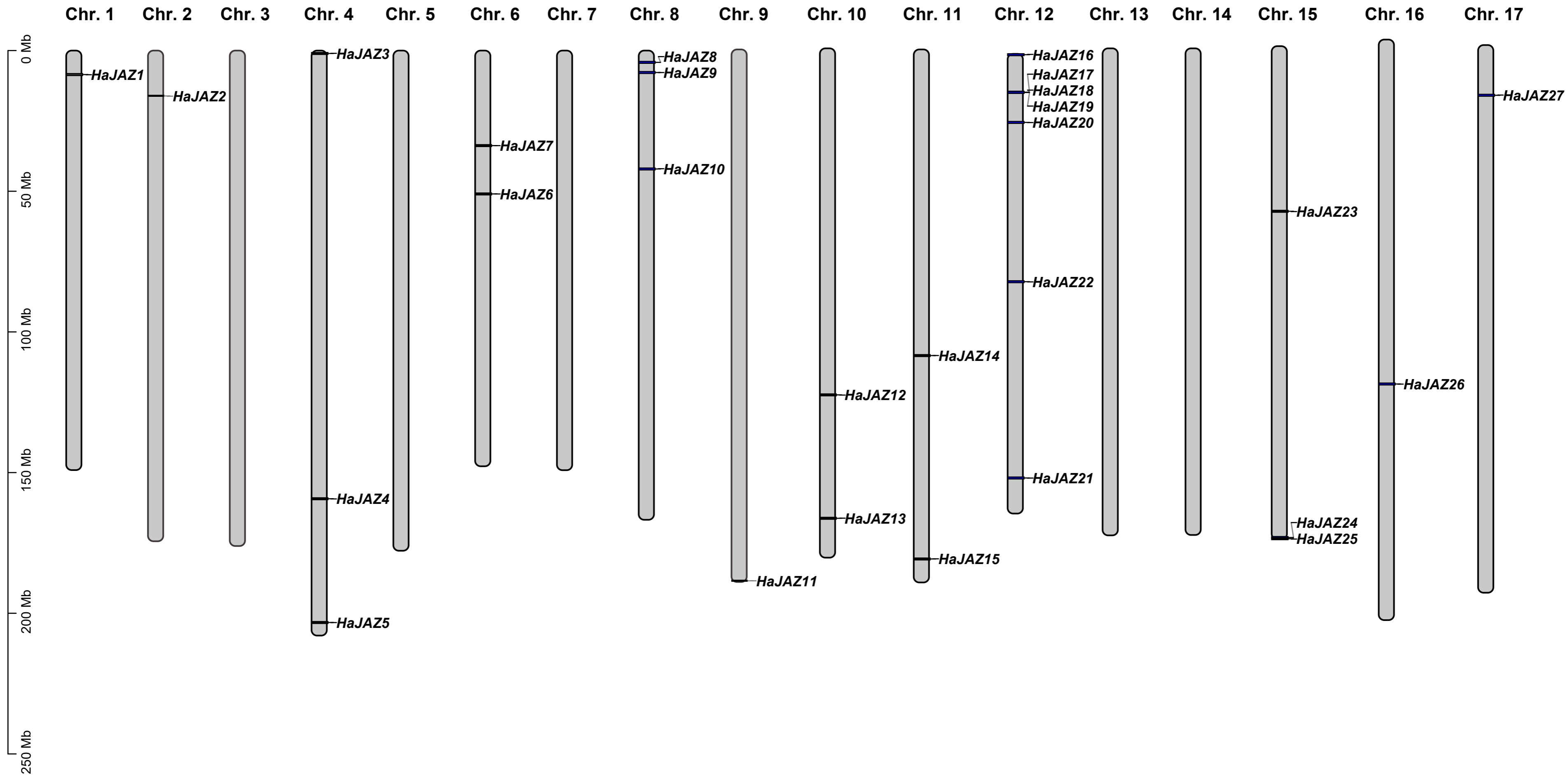

Supplement: SUPPLEMENTARY FIGURE S1 — Chromosomal locations of sunflower JAZ genes. The HaJAZ members are named according to their physical position on the chromosomes. [file Data_Sheet_1.PDF]

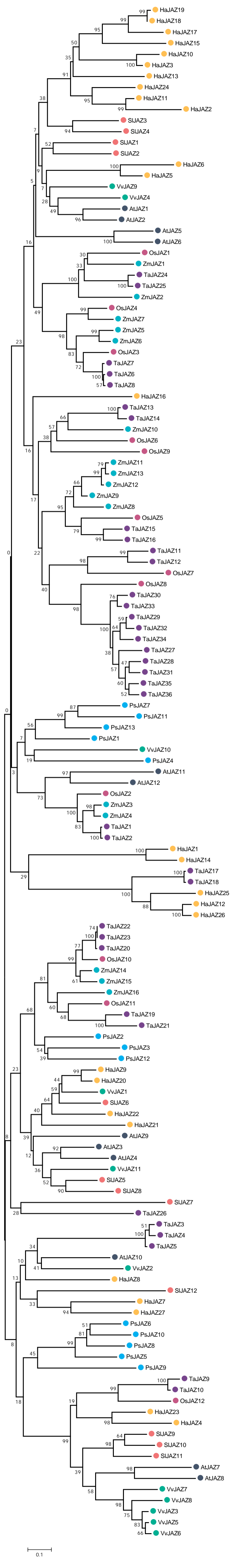

Supplement: SUPPLEMENTARY FIGURE S2 — The tree was constructed using amino acid sequences under neighbor-joining (NJ) methods in MEGA 7.0. The numbers on the nodes indicate the bootstrap values derived from 1,000 replicates performed to test the reliability; values <70% are not shown. [file Data_Sheet_2.PDF]

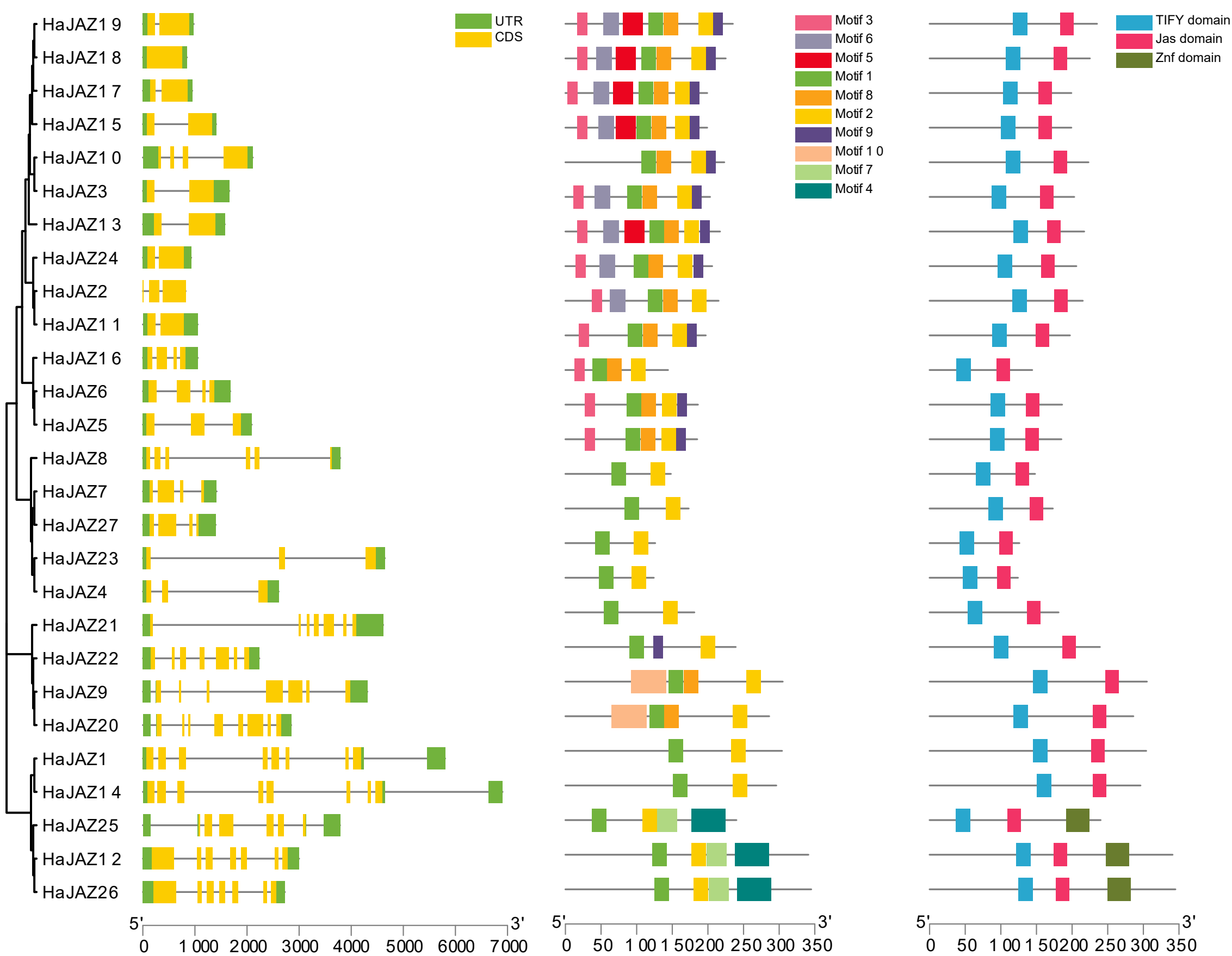

Supplement: SUPPLEMENTARY FIGURE S3 — Conserved gene structures, motif and domain distribution of the sunflower JAZ gene family. The gene structure graph was drawn based on the genome annotation results. The MEME online tool was used to predict the motifs and identify the ten motifs present in the JAZ protein sequence. The domain distributions were predicted with the SMART online tool. [file Data_Sheet_3.PDF]

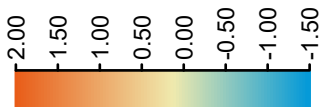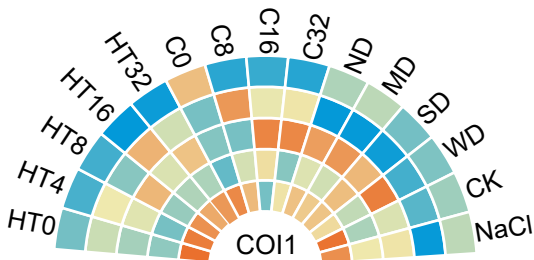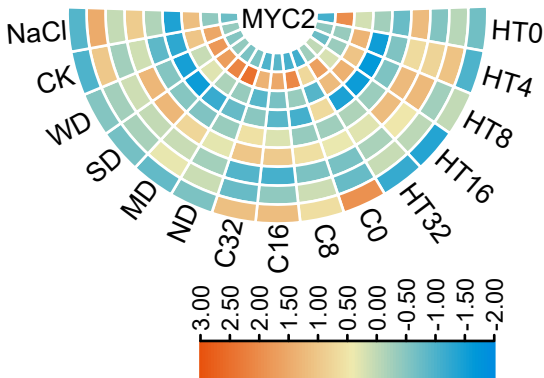

Supplement: SUPPLEMENTARY FIGURE S4 — The expression profiles of COI1 and MYC2 genes in response to abiotic stress treatments. The abbreviation name for each sample is shown in Figure 6E. [file Data_Sheet_4.PDF]
